# Chronic Obstructive Pulmonary Disease Mortality by Industry and Occupation — United States, 2020

**DOI:** 10.15585/mmwr.mm7149a3

**Published:** 2022-12-09

**Authors:** Girija Syamlal, Laura M. Kurth, Katelynn E. Dodd, David J. Blackley, Noemi B. Hall, Jacek M. Mazurek

**Affiliations:** 1 Respiratory Health Division, National Institute for Occupational Safety and Health, CDC.

Chronic obstructive pulmonary disease (COPD), a progressive lung disease, is characterized by long-term respiratory symptoms and airflow limitation (*1*). COPD accounts for most of the deaths from chronic lower respiratory diseases, the sixth leading cause of death in the United States in 2020.* Workplace exposures and tobacco smoking are risk factors for COPD; however, one in four workers with COPD have never smoked (*2*–*4*). To describe COPD mortality among U.S. residents aged ≥15 years categorized as ever-employed (i.e., with information on their usual industry and occupation), CDC analyzed the most recent 2020 multiple cause-of-death data^†^ from 46 states and New York City.^§^ Among 3,077,127 decedents, 316,023 (10.3%) had COPD^¶^ listed on the death certificate. The highest age-adjusted** COPD death rates per 100,000 ever-employed persons were for females (101.3), White persons (116.9), and non-Hispanic or Latino (non-Hispanic) persons (115.8). The highest proportionate mortality ratios (PMRs)^††^ were for workers employed in the mining industry (1.3) and in food preparation and serving related occupations (1.3). Elevated COPD mortality among workers in certain industries and occupations underscores the importance of targeted interventions (e.g., reduction or elimination of COPD-associated risk factors, engineering controls, and workplace smoke-free policies) to prevent COPD from developing and to intervene before illness becomes symptomatic or severe.

The analysis included 3,077,127^§§^ U.S. residents aged ≥15 years from 47 jurisdictions (46 states and New York City) who died during 2020 and whose record in National Vital Statistics System public use multiple-cause-of-death data included information on their usual^¶¶^ industry and occupation. COPD was identified using the *International Classification of Diseases, Tenth Revision* (ICD-10*)* codes J40–J44 listed as the underlying or contributing cause of death. The 23 two-digit industries and 26 occupations were grouped according to the 2012 North American Industry Classification System and the 2010 Standard Occupational Classification, respectively.***

Death rates (per 100,000 ever-employed persons) were based on postcensal population estimates as of July 1, 2020. Death rates were age-adjusted to the 2000 U.S. population. PMRs adjusted for age, sex, and race were calculated. A PMR with the lower 95% CI >1.0 indicated a significantly higher proportion of deaths associated with COPD in a specified industry or occupation than expected. CIs were calculated assuming Poisson distribution of data. Analyses were conducted using SAS software (version 9.4; SAS Institute).

Among the 3,077,127 decedents, 316,023 (10.3%; age-adjusted death rate = 102.5 deaths per 100,000 ever-employed persons) had COPD listed on their death certificates as the underlying or contributing cause of death. The highest age-specific COPD death rate (855.8 deaths per 100,000 ever-employed persons) was for persons aged ≥75 years (Table 1). The highest age-adjusted death rates were for females (101.3), White persons (116.9), and non-Hispanic persons (115.8) (Table 1).

**TABLE 1:** Number, percentage, and rates of deaths for chronic obstructive pulmonary disease* among ever-employed^†^ persons aged ≥15 years, by selected characteristics — 46 states and New York City, 2020

Characteristic	No. of deaths from all causes^§^	COPD		
		No. of deaths (%)	Death rate	
			Unadjusted^¶^	Age-adjusted** (95% CI)
**Total**	**3,077,127**	**316,023 (10.3)**	**126.2**	**102.5 (102.5–102.9)**
**Age group, yrs**				
15–24	31,993	47 (0.1)	0.1	—
25–34	66,292	222 (0.3)	0.5	—
35–44	94,924	1,276 (1.3)	3.3	—
45–54	173,980	7,807 (4.5)	20.8	—
55–64	402,215	41,234 (10.3)	104.4	—
64–74	617,183	82,037 (13.3)	271.7	—
≥75	1,690,540	183,400 (10.8)	855.8	—
**Sex**				
Female	1,471,005	153,716 (10.4)	120.0	101.3 (100.3–101.8)
Male	1,606,122	162,307 (10.1)	132.7	99.4 (98.5–99.9)
**Race^††^**				
American Indian or Alaska Native	22,406	2,109 (9.4)	65.6	41.5 (37.5–45.5)
Asian or other Pacific Islander	96,981	4,721 (4.9)	26.9	16.3 (15.4–17.3)
Black or African American	408,549	27,422 (6.7)	84.0	62.1 (60.5–63.7)
White	2,549,191	281,771 (11.1)	143.8	116.9 (116.1–17.7)
**Ethnicity**				
Hispanic or Latino	277,756	13,934 (5.0)	32.4	21.1 (20.4–21.9)
Non-Hispanic or Latino	2,799,371	302,089 (10.8)	145.3	115.8 (115.0–116.6)
COPD*				
Chronic bronchitis	—	1,702	0.7	0.7 (0.5–0.7)
Emphysema	—	18,129	7.2	5.9 (5.7–6.0)
Other COPD	—	298,419	119.2	96.8 (96.1–97.4)

**Source:** National Vital Statistics System public use multiple cause files 2020. https://www.cdc.gov/nchs/data_access/vitalstatsonline.htm#Mortality_Multiple

**Abbreviations:** COPD = chronic obstructive pulmonary disease; ICD-10 = *International Classification of Diseases, Tenth Revision*.

* Decedents with COPD (ICD-10 codes J40–J44) listed as the underlying or contributing cause-of-death.

^†^ Decedents with information on their usual industry and occupation.

^§^ Among ever-employed U.S. residents aged ≥15 years from 47 jurisdiction (excluding Arizona, District of Columbia, Iowa, North Carolina, and Rhode Island) with information on their usual industry and occupation information.

^¶^ Death rates are per 100,000 workers, based on 2020 estimates released by U.S. Census Bureau on July 27, 2021. https://www.census.gov/programs-surveys/popest/technical-documentation/methodology.html; https://wonder.cdc.gov/single-race-population.html

** Age-adjusted death rates (per 100,000 workers) were calculated by applying age-specific death rates to the 2000 U.S. Census Bureau standard population age distribution. https://wonder.cdc.gov/wonder/help/mcd.html#Age-AdjustedRates

^††^ Race and Hispanic origin are reported separately on the death certificate. The American Indian or Alaska Native race category includes North, Central, and South American Indians, Eskimos, and Aleuts. The Asian or other Pacific Islander race category includes Chinese, Filipino, Hawaiian, Japanese, and other Asian or Pacific Islanders.

PMRs were significantly elevated among ever-employed persons in 10 of the 23 industries and 11 of the 26 occupations (Table 2) (Table 3). The three industries with the highest PMRs were mining (1.33), accommodation and food services (1.28), and construction (1.23). The three occupations with the highest PMRs were food preparation and serving related (1.30), healthcare support (1.29), and construction and extraction (1.29).

**TABLE 2:** Number and percentage of deaths from chronic obstructive pulmonary disease* and proportionate mortality ratio^†^ among ever-employed^§^ persons aged ≥15 years, by industry –– 46 states and New York City, 2020

Industry^¶^	No. of deaths from all causes**	COPD	
		No. of deaths (%)	PMR (95% CI)
Agriculture, forestry, fishing, and hunting	68,502	7,768 (11.3)	1.03 (1.00–1.05)^††^
Mining	22,706	3,275 (14.4)	1.33 (1.28–1.38)^††^
Utilities	30,236	3,209 (10.6)	0.96 (0.92–0.99)
Construction	224,353	26,673 (11.9)	1.23 (1.21–1.24)^††^
Manufacturing	386,796	43,509 (11.2)	1.04 (1.03–1.05)^††^
Wholesale trade	28,331	2,823 (10.0)	0.93 (0.90–0.97)
Retail trade	220,233	22,836 (10.4)	0.99 (0.98–1.01)
Transportation and warehousing	161,208	18,745 (11.6)	1.14 (1.12–1.16)^††^
Information	55,556	5,207 (9.4)	0.86 (0.84–0.88)
Finance and insurance	88,115	7,780 (8.8)	0.80 (0.79–0.82)
Real estate and rental and leasing	37,290	3,601 (3.6)	0.89 (0.87–0.92)
Professional, scientific, and technical services	113,239	9,060 (8.0)	0.75 (0.73–0.76)
Management of companies and enterprises	4,587	462 (10.1)	0.89 (0.82–0.97)
Administrative, support, and waste management and remediation services	71,284	6,798 (9.5)	1.04 (1.02–1.07)^††^
Education services	203,542	14,954 (7.3)	0.68 (0.67–0.68)
Healthcare and social assistance	266,570	26,857 (1.1)	1.00 (0.99–1.01)
Arts, entertainment, and recreation	40,380	3,916 (9.7)	0.98 (0.95–1.02)
Accommodation and food services	114,117	12,721 (11.1)	1.28 (1.25–1.30)^††^
Other services (except public administration)	145,870	15,073 (10.3)	1.03 (1.02–1.05)^††^
Public administration	145,493	14,511 (10.0)	0.92 (0.91–0.94)
Military	31,044	4,145 (13.4)	1.23 (1.19–1.27)^††^
Other-misc, missing	617,675	62,100 (13.4)	1.02 (1.01–1.03)^††^

**Source:** National Vital Statistics System (NVSS) public use multiple cause files 2020. https://www.cdc.gov/nchs/data_access/vitalstatsonline.htm#Mortality_Multiple

**Abbreviations:** COPD = chronic obstructive pulmonary disease; ICD-10 = *International Classification of Diseases, Tenth Revision*; misc = miscellaneous; PMR = proportionate mortality ratio.

* Decedents with COPD (ICD-10 codes J40–J44) listed as the underlying or contributing cause of death.

^†^ PMR was defined as the observed number of deaths from COPD in a specified industry, divided by the expected number of deaths from COPD. The expected number of deaths was the total number of deaths in an industry of interest multiplied by a proportion defined as the number of COPD deaths in all industries, divided by the total number of deaths in all industries. PMRs were adjusted for 10-year age group, sex, and race.

^§^ Decedents with information on their usual industry and occupation.

^¶^ Industry the decedent worked in “during most of his or her life, or for the longest time” and is the two-digit simple industry recode based on the 2012 North American Industry Classification System–informed codes obtained from the U.S. Census Bureau. https://www.cdc.gov/nchs/data/dvs/Industry-and-Occupation-data-mortality-2020.pdf

** Ever-employed aged ≥15 years with information on their usual industry and occupation, information from 47 jurisdictions (excluding Arizona, District of Columbia, Iowa, North Carolina, and Rhode Island).

^††^ Significantly elevated PMR.

**TABLE 3:** Number, percentage of deaths from chronic obstructive pulmonary disease* and proportionate mortality ratio^†^ among ever-employed^§^ persons aged ≥15 years, by occupation –– 46 states and New York City, 2020

Occupation^¶^	No. of deaths from all causes**	COPD	
		No. of deaths (%)	PMR (95% CI)
Management	254,603	24.301 (9.5)	0.87 (0.86–0.88)
Business and financial operations	76,100	6,622 (8.7)	0.80 (0.79–0.82)
Computer and mathematical	25,320	1,803 (7.1)	0.71 (0.69–0.74)
Architecture and engineering	70,332	5,693 (8.1)	0.71 (0.70–0.73)
Life, physical, and social science	20,039	1,448 (7.2)	0.66 (0.63–0.68)
Community and social services	42,143	3,088 (7.3)	0.70 (0.68–0.72)
Legal	18,257	1,312 (7.2)	0.64 (0.62–0.67)
Education, training, and library	133,542	8,705 (6.5)	0.60 (0.59–0.61)
Arts, design, entertainment, sports, and media	47,606	4,047 (8.5)	0.82 (0.80–0.84)
Healthcare practitioners and technical	116,891	10,734 (9.2)	0.88 (0.86–0.89)
Healthcare support	52,528	6,281 (12.0)	1.29 (1.25–1.32)^††^
Protective service	54,826	5,721 (10.4)	1.02 (1.00–1.04)
Food preparation and serving related	91,368	10,315 (11.3)	1.30 (1.27–1.33)^††^
Building and grounds cleaning and maintenance	95,098	9,718 (10.2)	1.10 (1.07–1.12)^††^
Personal care and service	67,952	7,267 (10.7)	1.11 (1.08–1.14)^††^
Sales and related	214,771	21,705 (10.1)	0.94 (0.93–0.96)
Office and administrative support	272,811	27,265 (10.0)	0.93 (0.92–0.94)
Farming, fishing, and forestry	22,299	2,595 (11.6)	1.14 (1.09–1.19)^††^
Construction and extraction	206,217	25,730 (12.5)	1.29 (1.27–1.31)^††^
Installation, maintenance, and repair	106,146	13,120 (12.4)	1.20 (1.17–1.22)^††^
Production	240,443	28,247 (11.7)	1.11 (1.09–1.12)^††^
Transportation and material moving	214,521	25,389 (11.8)	1.22 (1.20–1.24)^††^
Military	30,360	4,046 (13.3)	1.22 (1.18–1.27)^††^
Other-misc (except housewife)	215,532	20,513 (9.5)	1.14 (1.12–1.16)^††^
Other-housewife	387,422	40,358 (10.4)	0.96 (0.96–0.97)

**Source:** National Vital Statistics System (NVSS) public use multiple cause files 2020. https://www.cdc.gov/nchs/data_access/vitalstatsonline.htm#Mortality_Multiple

**Abbreviations:** COPD = chronic obstructive pulmonary disease; ICD-10 = *International Classification of Diseases, Tenth Revision*; misc = miscellaneous; PMR = proportionate mortality ratio.

* Decedents with COPD (ICD-10 codes J40–J44) listed as an underlying or contributing cause-of-death.

^†^ PMR was defined as the observed number of deaths from COPD in a specified occupation, divided by the expected number of deaths from COPD. The expected number of deaths was the total number of deaths in an occupation of interest multiplied by a proportion defined as the number of COPD deaths in all industries, divided by the total number of deaths in all industries. PMRs were adjusted for 10-year age group, sex, and race.

^§^ Decedents with information on their usual industry and occupation.

^¶^ Occupation the decedent worked in “during most of his or her life, or for the longest time” and is the two-digit simple occupation recode based on the 2010 Standard Occupation Classification–informed codes obtained from the U.S. Census Bureau. https://www.cdc.gov/nchs/data/dvs/Industry-and-Occupation-data-mortality-2020.pdf

** Ever-employed persons aged ≥15 years with information on their usual industry and occupation information from 47 jurisdictions (excluding Arizona, District of Columbia, Iowa, North Carolina, and Rhode Island).

^††^ Significantly elevated PMR.

## Discussion

In 2020, 10% of deaths among ever-employed persons aged ≥15 years in 47 jurisdictions were associated with COPD. Elevated age-adjusted COPD death rates among White and non-Hispanic persons^†††^ are consistent with previous findings of increased COPD morbidity and mortality among these groups (*3*,*5*). During 2012–2018, an estimated 5.8 million (annual average) currently employed U.S. workers had COPD (*3*). An estimated 40% of adults with COPD have never smoked, and an estimated 24% of all COPD cases among never-smokers were attributed to workplace exposures (*2*–*4*), including dust, fumes, gases, vapors, and secondhand smoke (*2*). To reduce the prevalence of COPD among workers, the COPD National Action Plan^§§§^ emphasizes that occupational risk factors and interventions should be included in messaging and communication campaigns. In addition, COPD should be incorporated into prevention programs that address occupational risk factors.^¶¶¶^ Higher proportions of COPD deaths were observed for ever-employed persons whose usual industry was mining, accommodation and food services, construction, or transportation and material moving, and among workers whose usual occupation was healthcare support, food preparation and serving related, construction and extraction, or transportation and material moving. National survey data indicates that workers in these industries and occupations have elevated prevalence of COPD, higher tobacco use, and are frequently exposed to secondhand smoke, vapors, gas, dust, and fumes in the workplace (*2*,*3*,*6*–*8*). For example, approximately one third of the workers in mining, construction, accommodation and food services, and transportation and warehousing industries, and healthcare support, construction and extraction, food preparation and serving related occupations are combustible tobacco users and are often exposed to secondhand smoke, diesel exhaust, and byproducts of machinery combustion, as well as dusts (e.g., wood and silica dusts), vapors, and fumes (*6*–*8*). In addition, a previous study among nurses and healthcare support workers found that exposure to cleaners and disinfectants (i.e., glutaraldehyde, bleach, hydrogen peroxide, alcohol, and ammonium compounds) was associated with increased (25%–38%) risk for COPD (*9*).

Although the exact reason for the differences in high COPD death rates among certain groups is unknown, differences could be partly explained by preventable workplace exposures including secondhand smoke, vapors, dusts, and fumes (*2*,*6*,*8*). Identification of hazards in the workplace could assist with early identification and implementation of public health programs (e.g., workplace smoke-free policies and cessation programs, elimination or substitution of exposures, removing workers from exposures, and engineering controls such as ventilation or enclosure of exposure-generating processes) that support comprehensive approaches to prevention through control of workplace hazards and promotion of healthy behaviors, early interventions, and better access to health care services (*8*).

The findings in this report are subject to at least six limitations. First, COPD-related deaths were not validated using medical records. Second, no information on workplace exposures is available on death certificates. Therefore, whether workplace exposures could have led directly to the COPD death is unknown. Third, if COPD was caused by workplace exposures, the industry and occupation information reported on the death certificate might not be the industry and occupation in which workplace exposures occurred. Fourth, 38,264 decedents (1.2% of total deaths) for whom employment history was not available on the death certificate were excluded from the current study. Fifth, information on smoking status of decedents was not available; smoking is known to cause or worsen COPD. Finally, results are limited to 47 jurisdictions and might not be representative of nonparticipating jurisdictions.

Findings from this report might help physicians identify workers who should be evaluated for COPD in the industries and occupations with a higher proportion of COPD deaths. The elevated COPD mortality among ever-employed persons in certain industries and occupations underscores the importance of targeted interventions to prevent COPD from developing and intervening before it becomes symptomatic or severe. Continued surveillance, including collection of detailed industry and occupational history and etiologic research to further characterize occupational risk factors for COPD, is essential to guide interventions and policies to improve workers’ health.

**Summary**

**What is already known about this topic?**

Chronic obstructive pulmonary disease (COPD) was the sixth leading cause of death in the United States in 2020. Workplace exposures and tobacco smoking are risk factors for COPD.

**What is added by this report?**

In 2020, 316,023 (10.3%) deaths among ever-employed persons were associated with COPD. The COPD proportionate mortality ratios were elevated for several industries and occupations, and highest among workers in the mining industry and in food preparation and serving–related occupations.

**What are the implications for public health practice?**

Elevated COPD mortality among workers in certain industries and occupations underscores the importance of targeted interventions, including reduction or elimination of COPD-related risk factors and workplace smoke-free policies, to prevent COPD from developing and to intervene before illness becomes symptomatic or severe.

## Footnotes

*
https://www.cdc.gov/nchs/fastats/leading-causes-of-death.htm

†
https://www.cdc.gov/nchs/nvss/mortality_public_use_data.htm

§ Forty-six states and New York City participated in a collaborative (National Center for Health Statistics [NCHS] and the National Institute for Occupational Safety and Health) program to translate industry and occupation information on death certificates to U.S. Census Bureau Industry and Occupation codes (https://www.cdc.gov/nchs/data/dvs/Industry-and-Occupation-data-mortality-2020.pdf). Arizona, District of Columbia, North Carolina, and Rhode Island did not participate. Because of differences in collection methods, Iowa’s data were not consistent with those from other states and were excluded.

¶ ICD-10 codes J40 (bronchitis, not specified as acute or chronic), J41 (simple mucopurulent chronic bronchitis), J42 (unspecified chronic bronchitis), J43 (emphysema), and J44 (other chronic obstructive pulmonary disease), assigned as the underlying (the disease or injury that initiated the chain of events that led directly and inevitably to death) or contributing cause of death. https://wonder.cdc.gov/; https://www.cdc.gov/nchs/icd/icd10.htm

** Age-adjusted death rates were calculated by applying age-specific death rates to the 2000 U.S. Census Bureau standard population age distribution. https://wonder.cdc.gov/wonder/help/mcd.html#Age-Adjusted

†† PMR was defined as the observed number of deaths from COPD in a specified industry or occupation, divided by the expected number of deaths from COPD. The expected number of deaths was the total number of deaths in the industry or occupation of interest multiplied by a proportion defined as the number of COPD deaths in all industries or occupations, divided by the total number of deaths in all industries or occupations. The COPD PMRs were adjusted by 10-year age groups, sex, and race. A PMR >1.0 indicates that more deaths were associated with the condition in a specified occupation or industry than expected. https://wwwn.cdc.gov/eworld/Appendix/Mortality

§§ NCHS multiple cause-of-death data included 3,390,278 decedents. Foreign residents, decedents aged <15 years, persons with missing age, and decedents from the five nonparticipating or excluded jurisdictions were not included in industry and occupation coding (3,115,391). Decedents whose death certificate lacked information on industry and occupation (38,264) were excluded from the analysis, resulting in 3,077,127 records.

¶¶ Usual occupation (and corresponding business or industry) is not necessarily the occupation of the decedent at the time of death, but the occupation the person did for “most of his or her working life,” based on funeral director’s discussions with decedent’s informant. https://www.cdc.gov/niosh/topics/noms/funeral.html; https://www.cdc.gov/niosh/docs/2012-149/default.html

***
https://www.cdc.gov/nchs/data/dvs/Industry-and-Occupation-data-mortality-2020.pdf

††† https://www.lung.org/research/trends-in-lung-disease/copd-trends-brief/data-tables/trends-in-mortality-sex-and-race; https://www.lung.org/research/trends-in-lung-disease/copd-trends-brief/copd-prevalence

¶¶¶
https://www.nhlbi.nih.gov/resources/copd-national-action-plan
